# An Evaluation of the Anti-Rabies Effect of Bufotenine in Murine Rabies Models to Determine Its Mechanism of Action

**DOI:** 10.3390/v17060808

**Published:** 2025-05-31

**Authors:** Patrícia Mariano Cruz Pereira, Andréa de Cássia Rodrigues Silva, Karen Miyuki Asano, Adriana da Costa Neves, Juliana Mozer Sciani, Daniel Carvalho Pimenta, Hugo Vigerelli

**Affiliations:** 1 Laboratory of Biochemistry and Biophysics, Butantan Institute, São Paulo 05503-900, SP, Brazil; patricia.cruz@fundacaobutantan.org.br (P.M.C.P.); hugo.barros@butantan.gov.br (H.V.); 2 Programa de Pós-Graduação em Toxinologia, Butantan Institute, São Paulo 05503-900, SP, Brazil; juliana.sciani@usf.edu.br; 3 Laboratory of Rabies Diagnostic, Serology, Pasteur Institute, São Paulo 01311-000, SP, Brazil; arsilva@pasteur.saude.sp.gov.br (A.d.C.R.S.); kmasano@pasteur.saude.sp.gov.br (K.M.A.); 4 Laboratory of Genetics, Butantan Institute, São Paulo 05503-900, SP, Brazil; adriana.neves@butantan.gov.br; 5 Laboratory of Natural Products, Bragança Paulista 12916-900, SP, Brazil; 6 Laboratory of Ecology and Evolution (LEEV), Butantan Institute, São Paulo 05503-900, SP, Brazil; 7 Centre of Excellence in New Target Discovery (CENTD), Butantan Institute, São Paulo 05503-900, SP, Brazil

**Keywords:** rabies virus, alkaloid, bufotenine, immune system, antiviral

## Abstract

Molecules from animals or plant species have been investigated with the aim of treating diseases of epidemiological importance, such as rabies, which is a viral, acute, and infectious disease with approximately 100% lethality. Rabies has been one of the main causes of death in humans concerning infectious diseases. This work investigated the action and preliminary mechanisms of the alkaloid bufotenine in an in vivo model with the rabies virus. A wild-type virus was titrated and injected into mice for the determination of DL50 in the presence or absence of bufotenine. The results reveal that bufotenine has possible action in modulating the immune response of the studied host, suggesting interference in delaying symptom manifestation. Regarding the histological analysis of the CNS of the animals, bufotenine possibly prevented the presence of mononuclear cell inflammatory infiltrate in the meninx’s region compared to the positive control and possibly contributed to reducing neuronal degeneration. The use of the bufotenine extracted from the seed of white angico, a plant representative of Brazilian flora, contributed to antiviral activity with effects on the immunological aspects of the host infected by the rabies virus.

## 1. Introduction

Rabies holds significant importance for public health due to the high number of occurrences, high mortality rate, and financial costs associated with treatments, adverse reactions to administered vaccines, and related diagnostics. Despite the availability of preventive vaccines, rabies has been among the top ten causes of death in humans due to infectious diseases. However, the World Health Organization (WHO) has classified it as a low priority compared to other diseases like tuberculosis and AIDS [1].

The rabies virus begins replication at the initial contact site, migrating through peripheral nerves towards the central nervous system (CNS), causing irreversible tissue and organ damage and ultimately leading to death. The host’s immune response starts only after clinical symptoms appear, with low levels of neutralizing antibodies observed throughout the infection, increasing only before death [2,3].

Despite the nearly 100% lethality of rabies, there have been two reported cases of recovery through the Milwaukee Protocol in the United States in 2004 and the Recife Protocol in Brazil in 2008. This treatment involves inducing the patient into a coma with antiviral medication to allow the body time to produce sufficient antibodies to combat the virus. However, the use of these protocols has not shown positive results in other cases, and the antiviral drugs used are not specific to the rabies virus. Research has indicated that Ribavirin, when combined with Ketamine and Amantadine, does not show satisfactory antiviral activity [4,5]. Thus, new treatments are necessary for the management and cure of this disease.

With the increase in studies on the antiviral action of new natural products, it has become possible to isolate and characterize new compounds that can inhibit viral replication or expand the database with new molecules [6]. In vitro biological systems facilitate drug discovery, while cell culture models help obtain toxicological profiles of compounds during testing phases. Thus, promising molecules are often discarded due to observed toxicity during cell culture assays [7]. In 2011, Smith et al. noted that the number of studies using potential antiviral agents increased for the rabies virus, but such compounds did not show reproducibility when subjected to animal model tests [8].

On the other hand, some natural compounds have shown important antiviral effects with low toxicity. Our research group has focused on this research: Vigerelli et al. (2014) pointed out that bufotenin, isolated from the cutaneous secretion of *Rhinella jimi* and seeds of *Anadenanthera colubrina*, inhibited rabies virus infection in mammalian cells (BHK-21) [9]. Cunha Neto et al. (2015) observed a synergistic effect between the natural antimicrobial peptide ocellatin-F1 (isolated from the cutaneous secretion of *Leptodactylus labyrinthicus*) and bufotenin against the rabies virus in BHK-21 cell culture [10]. Vigerelli et al. (2020) confirmed the in vitro antiviral effect of bufotenin on different rabies virus variants and its ability to prevent disease symptoms in 40% of infected mice compared to 15% in the control group [11].

Thus, in this study, we evaluated the effect of bufotenine in an in vivo model of rabies on the reduction in virus titer in the CNS and the participation of the immunological system in the elimination of viruses.

## 2. Materials and Methods

### 2.1. Bufotenine Attainment

Bufotenine was obtained from the seeds of *Anadenanthera colubrina*, obtained from the supplier Arbocenter Comércio de Sementes Ltda, Birigui, São Paulo, batch 0019. The seeds were triturated, and a liquid–liquid partition technique was performed by mixing 1 mL of sample with 1 mL of ultrapure water in a test tube. Subsequently, 1 mL of dichloromethane was added to the tube, which was then centrifuged to ensure more effective phase separation. The phase containing dichloromethane was evaporated under a hood for 24 h to prevent the appearance of solvent peaks when subjected to reverse-phase high-performance liquid chromatography (RP-HPLC). The phases were separated into properly labeled tubes and stored at −20 °C.

The product was fractionated in a binary HPLC system (20A Prominence, Shimadzu Co., Kyoto, Japan), where the samples were injected into a C18 column (ACE^®^ 250 × 4.6 mm) using solvents (A) trifluoroacetic acid/H_2_O (1:1000) and (B) trifluoroacetic acid/acetonitrile/H_2_O (1:900:100) at a constant flow rate of 1 mL/min at 30 °C. The gradient used was 10 to 70% of solvent B over 40 min for solution A. The fractions resulting from the process were collected in separate tubes and concentrated using a vacuum concentration system for subsequent mass spectrometry analyses. The yield in grams of each peak obtained was weighed and stored in a freezer at −20 °C until use.

The peak correspondent to bufotenine was analyzed by mass spectrometry, conducted using an ESI-IT-Tof (Shimadzu Co., Kyoto, Japan). The samples were diluted in 50% acetonitrile in water containing 0.5% acetic acid and injected directly into the spectrometer via automatic injection in positive mode, with a flow rate of 50 μL/min, using the same solution for sample dilution. The interface voltage used was 4.5 kV, and the detector voltage was 1.76 kV, with a temperature of 200 °C. Fragmentation was carried out with argon collision gas, using 50% energy, and spectra were obtained in the range of 50 to 2000 *m*/*z*. The obtained data were analyzed using LCMSsolution v3.81 software (Shimadzu Co., Kyoto, Japan).

### 2.2. Rabies Virus Obtention

For the in vivo experimental assay, a sample from the Pasteur Institute’s sample bank was used, whose genetic lineage is compatible with domestic dog (IP1972/16). This sample originated from a dog (*Canis lupus familiaris*) in the municipality of São Luís, state of Maranhão, whose central nervous system (CNS) was sent to the Diagnostic Section of the Pasteur Institute in 2016, and it tested was positive for rabies. Additionally, the laboratory-fixed viruses PV (Pasteur Virus) and CVS (Challenge Virus Standard—CVS/31, ref. [1]) were also used.

### 2.3. Virus Production

Virus production was carried out in flasks containing 8 mL of MEM medium supplemented with FBS, 30 μL of antibiotic (gentamicin), and 30 μL of amino acids. After a period, the medium was replaced with DMEM medium, and amino acids were no longer required. Subsequently, 1 mL of resuspended N_2_A cells and 1 mL of thawed isolated viruses, previously defrosted on ice, were added and incubated for 72 h. After this period, the viral suspension was collected and centrifuged at 3000 rpm at 4 °C for 10 min. Following centrifugation, the supernatants were collected and stored in a freezer at −80 °C.

After viral production, dilution was performed in 96-well microplates on ice. Dilutions were established in duplicates. In both duplicates, the virus was diluted to a ratio of 1:2 in the first well (50 μL of medium + 50 μL of the produced sample), and serial dilutions followed at a 2-fold ratio until the twelfth well. Subsequently, with the aid of an automatic micropipette, the first well of both duplicates was homogenized to transfer 50 μL of the content to the next well, and so on, until reaching the twelfth well, where 50 μL was discarded. Next, 150 μL of MEM supplemented with 10% fetal bovine serum and 100 μL of resuspended N2A cells were added to all wells, and the plate was incubated in an oven at 37 °C with a 5% CO_2_ atmosphere for 96 h. After this period, the plate was aspirated and kept on ice for cell fixation with 180 μL of 80% acetone, previously refrigerated [12]. The reaction was revealed with the addition of 40 μL of anti-rabies virus conjugate produced by the Pasteur Institute. The plates were then washed by immersion in PBS and distilled water, dried, and supplemented with 50 μL of 10% glycerin. The reading was performed on an inverted fluorescence microscope (Leica DMIL, 200× magnification).

### 2.4. Genetic Confirmation of Virus Produced

RNA was extracted using a suspension of 300 μL from produced viruses. For the negative control, 300 μL of ultrapure water free of DNase and RNase was used, and for the positive control, a 300 μL suspension of CVS/31 was used.

Total RNA extraction was performed using the TRIzol method (Invitrogen^TM^ Life Technologies, Carlsbad, CA, USA) following the manufacturer’s instructions. Resuspension was carried out with 50 μL of ultrapure water free of DNase/RNase. After extraction, the total RNA was stored in a freezer (−80 °C) until use.

Reverse transcription was carried out using SuperScript II reverse transcriptase (Invitrogen^TM^ Life Technologies, Carlsbad, CA, USA) according to the manufacturer’s instructions. The PCR assay was performed with forward primers (5′-ACGCTT AACAACAARATCARAG-3′) [13]) and reverse P784 (5′-CCTCAAAGTTCT TGTGGAAGA-3′) [14] using Taq DNA polymerase (Invitrogen^TM^ Life Technologies, Carlsbad, CA, USA) following the manufacturer’s instructions.

The PCR products were subjected to agarose gel electrophoresis (1%), and the amplicons were purified using the GFX PCR DNA and Gel Band Purification Kit (GE Healthcare, Buckinghamshire, UK).

Sequencing was performed using a Bigdye Terminator v3.1 Cycle Sequencing Kit (Applied Biosystems, Foster City, CA, USA). DNA sequences were determined using an ABI 3130 Genetic Analyzer (Applied Biosystems, Foster City, CA, USA).

The generated sequences were submitted to analysis through the Phred software (http://asparagin.cenargen.embrapa.br/phph/, accessed on 30 April 2025) and edited using the Bioedit program (Hall, 1999). Sequence identities were confirmed by comparing them with GenBank data through the NCBI BLASTn (Basic Local Alignment Search Tool) program (http://blast.ncbi.nlm.nih.gov/Blast.cgi, accessed on 30 April 2025), accessed on 28 July 2024.

### 2.5. Direct Immunofluorescence

The technique was performed to detect the viral antigen of virus sample, specifically to confirm positivity before and after the first inoculation in animals. Imprints of CNS fragments were prepared on slides according to the technique described by Dean et al. (1996) [15]. The conjugated antibody used was the polyclonal anti-rabies virus antibody. The slide readings were carried out with the aid of 90% glycerin (with carbonate/bicarbonate buffer at pH 8) using a Leica DMBL fluorescence microscope (100×).

### 2.6. Virus Titration in Mice

The use of animals for the experiment was approved by the Animal Ethics Committee of the Pasteur Institute (CEUA-IP) (Protocol No.: 04.2019) and the Animal Ethics Committee of the Butantan Institute (CEUAIB) (Protocol No.: 4495171019).

Heterogeneous male and female mice from the Pasteur Institute of São Paulo’s animal facility (Swiss) aged 21 days and weighing between 11 and 14 g and mice aged 28 days and weighing between 18 and 24 g were used. The 21-day-old mice were employed for virus production, while the 28-day-old mice were used for virus titration (IP1972/16).

The animals were housed in appropriate cages in a climate-controlled room of the Pasteur Institute of São Paulo’s animal facility with a light/dark cycle (12 h/12 h). They were provided with a diet of commercially pelleted mouse feed ad libitum and filtered water in drinking bottles ad libitum. A maximum of five animals per cage was established.

Virus titration in mice was performed to determine the 50% lethal dose (LD50), using the method by Reed and Müench [16]. A single sample was titrated since only one strain was in the in vivo experimental assay (IP1972/16, a genetic strain compatible with domestic dogs/variant 2).

First, a 20% suspension composed of CNS (IP1972/16) and diluent solution (consisting of 0.85% saline, 2% fetal bovine serum, and 1 mL of gentamicin per liter) was inoculated into 6 individuals (21-day-old Swiss mice) intracerebrally (0.03 mL) to obtain a sufficient volume for conducting the entire animal experiment. After the disease progressed to an advanced stage, the animals were euthanized, and the CNS of each mouse was collected to prepare a single suspension, which was titrated and used in the in vivo experiment (Section 2.7).

For titration, 5 groups consisting of 8 animals each were initially established, resulting in 40 animals in total. Each group was inoculated with different dilutions (10^0^, 10^−1^, 10^−2^, 10^−3^, and 10^−4^). Inoculation was performed via a plantar pad, and the animals were evaluated daily over 50 days by recording their weights and clinical signs. They were euthanized after the advanced clinical disease stage was reached, using high doses of the inhalation anesthetic isoflurane. However, the presented results were obtained by referring to data observed up to the 36th observation day.

The clinical signs were tabulated according to the progression of the disease in the 1st (apathy, piloerection, and hunched posture), 2nd (asymmetric limb movement, partial prostration, tremors, and hindlimb paresis initially observed in the inoculated limb (left)), and 3rd stages (forelimb and hindlimb paresis, seizures, and complete prostration) of infection for each animal in the dilution groups.

LD50 was calculated based on the cumulative mortality rate [16].

### 2.7. In Vivo Experiments

For the in vivo experiment, Balb/c mice, 28 days old and weighing between 19 and 25 g, provided by the Butantan Institute’s animal facility were used. A dose equivalent to 10 LD_50_ was administered for the experimental procedure.

Four treatment groups were established, as detailed in Table 1. However, the data presented and discussed in this article pertain exclusively to the rabies control group and the rabies/bufotenine group. On day 0, all animals (the rabies control group and the rabies/bufotenine group) were inoculated with viral CNS suspension from sample IP1972/16 (a genetic lineage compatible with domestic dogs) in the plantar pad to simulate an accident.

One hour after viral inoculation, animals in the rabies group (*n* = 21) received a subcutaneous injection of 250 μL of saline (NaCl), while animals in the virus + bufotenine group (*n* = 21) received a subcutaneous injection of 250 μL of saline (NaCl) containing 0.63 mg of bufotenine. On subsequent days, the same treatments were maintained once daily.

On the first day of the experiment (day 0) and subsequent days, animals were weighed and observed according to the symptoms related to rabies accident.

Based on the titration results of the CNS from the IP1972/16 sample, three time points were established at 5-day intervals for euthanizing animals from each group. The selected days were the 5th, 10th, and 15th days post-inoculation.

After the experiment, the animals were properly anesthetized with inhaled isoflurane in an anesthetic chamber. Exsanguination was performed via cardiac puncture using syringes coated with 3% EDTA anticoagulant. After collecting blood from each animal, it was transferred to 2 mL tubes. Subsequently, all tubes were centrifuged for 10 min at 2500 rpm at 4 °C to separate the serum. One aliquot was designated for RFFIT, and the other for flow cytometry analysis. All materials were stored in a freezer at −20 °C until use.

In a biosafety cabinet, the CNSs and spinal cords of each animal were collected.

Brain collection was carried out using sterile scissors to make transversal, longitudinal, and lateral cuts that allowed for the removal of the cranial cap and the intact brain. After collection, the brains were transferred to properly labeled containers containing 10% formalin for preservation.

The other brains not designated for histology were collected, weighed, and separately macerated with a diluent solution to prepare their respective 20% suspensions. After maceration and transferring the contents to centrifuge tubes, these were placed in a refrigerator for 30 min to allow the antibiotic in the diluent to act. After this period, the tubes were centrifuged for 45 min. The suspensions obtained from each CNS were then subdivided into two 2 mL Eppendorf tubes. One tube was designated for CNS titration, and the other for flow cytometry. These tubes were stored in a −80 °C freezer.

Spinal cord collection was performed via hydraulic extraction by applying quick pressure with the plunger of a syringe containing water and a blunt-tip needle directed into the lumbar spinal canal over a disposable Petri dish.

#### 2.7.1. Rapid Fluorescent Focus Inhibition Test (RFFIT)

Serum from animals was initially inactivated in a water bath at 56 °C for 30 min. Then, 25 μL of each serum was placed into the wells of microplates. Subsequently, 50 μL of MEM medium for BHK-21 cells, containing FBS 10%, was added. A 2-fold serial dilution of the sera was then performed, with an initial dilution of 1:5.

The CVS/31 strain virus or SNC suspension (40 μL) was diluted in MEM according to its titer and kept on ice to prevent loss of potency. Then, 50 μL of the virus was added to all wells containing serum and MEM. The plates were placed in a CO_2_ incubator (5%) at 37 °C for incubation for 1.5 h to allow virus neutralization. After the incubation period, 50 μL of BHK-21 cells at a concentration of 2.5 × 10^4^ cells/mL were added to the wells, and the plates were returned to the incubator for 20 h.

The cells were then fixed on ice with 200 μL of 80% acetone for 15 min. The plates were revealed by adding 40 μL of anti-rabies virus conjugate, produced by the Pasteur Institute, diluted in PBS with Evans blue, and incubated for 1 h in the CO_2_ incubator. The plates were washed by immersion in PBS and distilled water, dried, and supplemented with 50 μL of 10% glycerin to facilitate reading under a fluorescence microscope (Leica DMIL, 200× magnification).

#### 2.7.2. Cytokine Quantification via CBA—Cytometric Bead Array

Cytokine quantification was performed on serum samples and CNS suspensions from animals. Cytokines were quantified in singlet from the supernatant, including Interleukin-2 (IL-2), Interleukin-4 (IL-4), Interleukin-6 (IL-6), Interleukin-10 (IL-10), Interleukin-17 A (IL-17 A), Interferon-γ (IFN-γ), and Tumor Necrosis Factor-α (TNF-α) using the Mouse Th1/Th2/Th17 kit (BD, San Jose, CA, USA) via flow cytometry using the BD FACSCanto™ system.

#### 2.7.3. Histology

The brains were sectioned longitudinally using scalpel blades and fixed in 10% formalin for 48 h. Each cerebral hemisphere was placed into properly labeled cassettes and transferred to a tissue processor for dehydration, clearing, and infiltration in paraffin. After being embedded in paraffin, 4–6 μm thick sections were prepared and stained with hematoxylin and eosin (HE) and cresyl violet. Microphotographs were taken using a Zeiss microscope.

### 2.8. Statistical Analysis

Statistical analyses were performed using the one-way ANOVA test with Tukey’s post hoc test, employing the software GraphPad^®^ Prism 7.0 (GraphPad Software Inc., La Jolla, CA, USA). Values of *p* < 0.05 were considered statistically significant.

## 3. Results

### 3.1. Bufotenine Isolation

**Figure 1:**
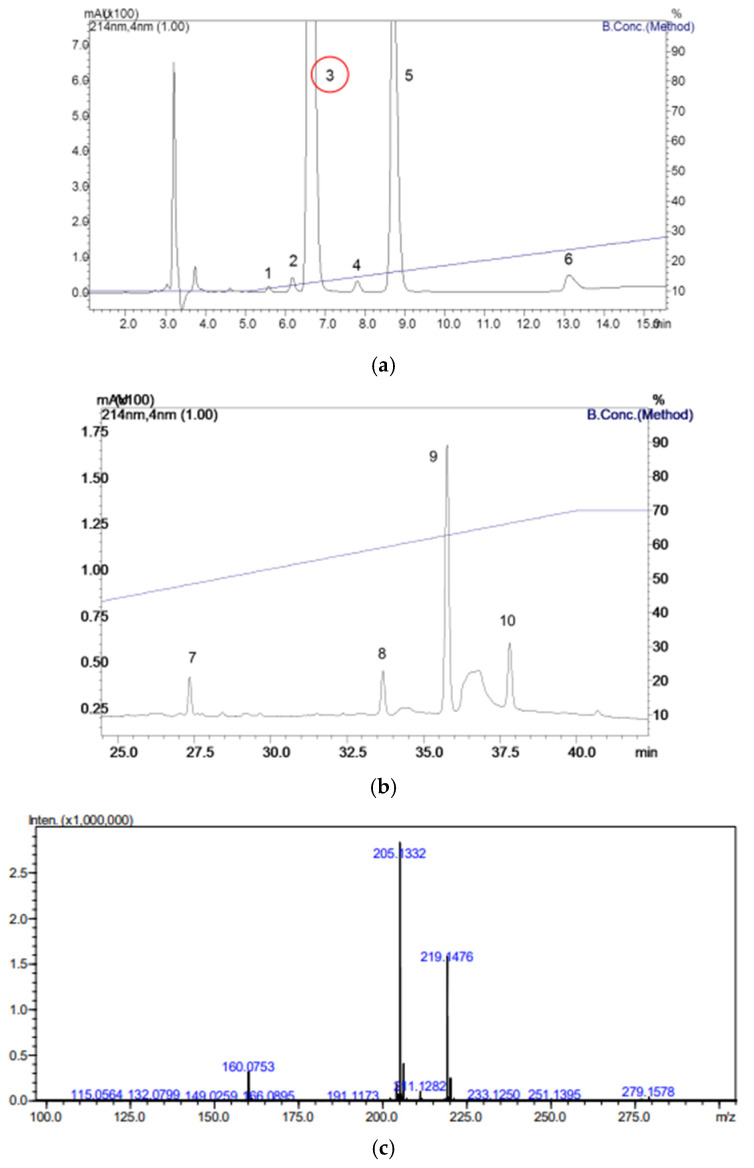
Chromatographic profile of *Rhinella schneideri* skin secretion obtained in C18 column and collection of peak 3, correspondent to bufotenine: (**a**) 0 to 15 min gradient; (**b**) 25 to 40 min gradient; (**c**) mass spectrometry analysis of peak 3 (highlighted in panel (**a**)).

Bufotenine was isolated from the cutaneous secretion of the amphibian *Rhinella schneideri*. A chromatographic profile, depicted in Figure 1a,b, shows ten distinct peaks. Peak 3, highlighted in red, was collected and analyzed by mass spectrometry. The mass spectrum exhibited characteristic ions at *m*/*z* 205 and 160, confirming the presence of bufotenine (Figure 1c).

### 3.2. Rabies Virus Obtention

**Figure 2:**
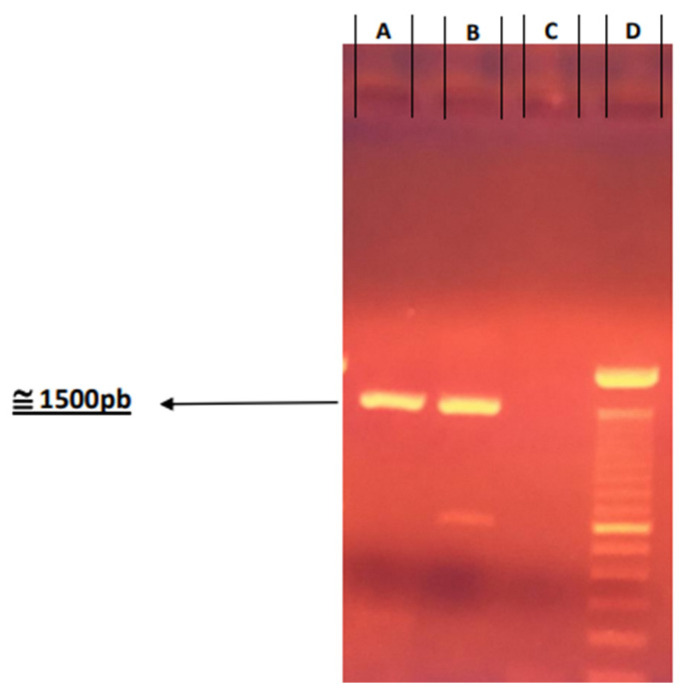
Agarose gel to show amplification of gene N (nucleoprotein from rabies virus). (A) IP1972/16 wide virus; (B) positive control CVS; (C) negative control (ultrapure water); (D) molecular mass marker.

A direct immunofluorescence test showed positive results for the IP1972/16 sample from the Pasteur Institute bank. This positivity was confirmed after a subsequent mouse inoculation to propagate the virus. Furthermore, viral isolation in cell culture provides additional evidence of virus presence. The IP1972/16 sample, used in subsequent assays, was conducted for sequencing analysis, which revealed genetic compatibility with domestic dogs’ lines. The agarose gel, depicted in Figure 2, demonstrates the presence of a band at the expected molecular weight for the N gene (1470 bp), which encodes the nucleoprotein of the rabies virus from street virus isolates.

### 3.3. Animal Model of Rabies

**Figure 3:**
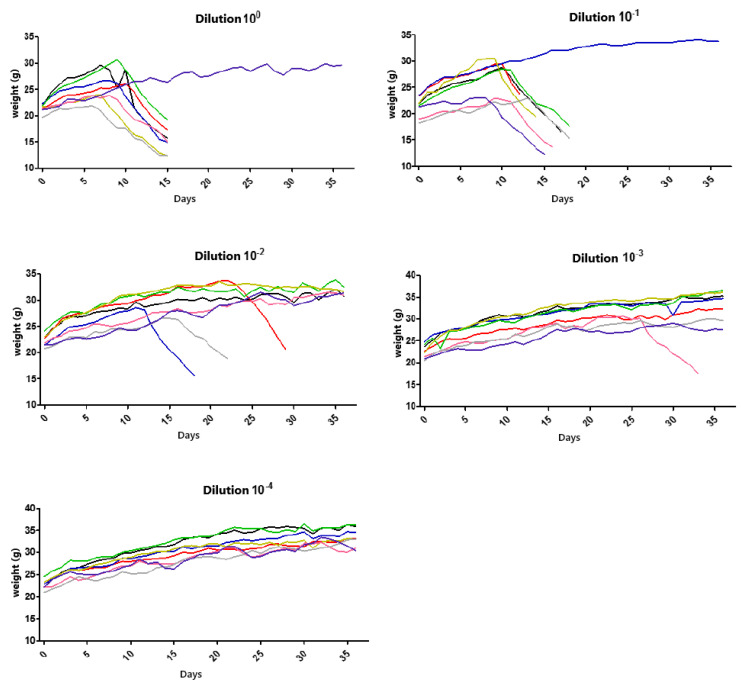
Weight (g) of mice by dilution group after 36 days of experiment. Each color represents one different test subject.

Animals were observed for 36 days after virus inoculation. The most evident clinical sign was weight loss, shown in Figure 3, which depicts several virus dilutions. It was observed that the animals’ weights significantly decreased after approximately 15 days, when exposed to the highest concentrations. In contrast, no weight change was noted in the animals treated with the 10^4^ dilution even after 36 days (Figure 3).

In addition to weight loss, clinical signs were observed in inoculated mice during the progression of the disease. In the first stage of infection, apathy, piloerection, and an arched posture were noticed. Asymmetrical limb movement, partial prostration, tremor, and initial paresis of the hind limb on the inoculated side (left) marked the second stage. The third stage of infection was characterized by paresis of both the fore and hind limbs, convulsions, and complete prostration.

**Figure 4:**
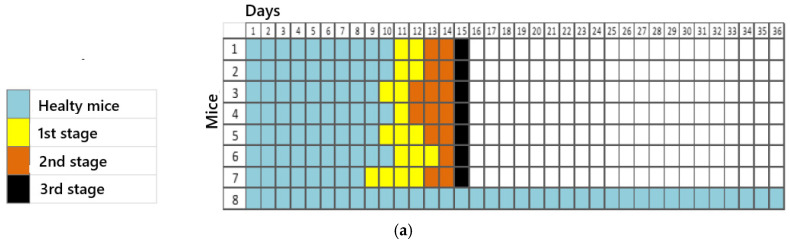

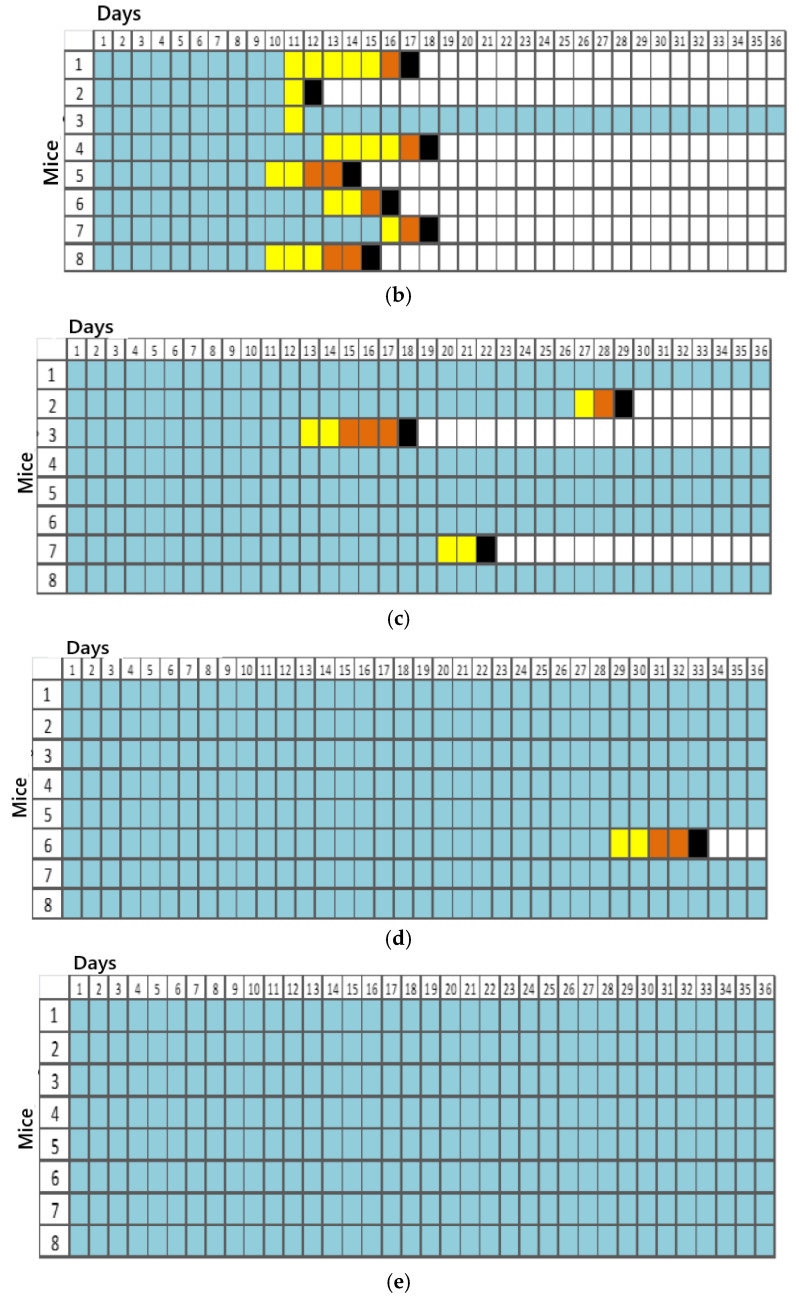
Clinical manifestation of the disease induced in mice, with the IP1972/16 virus model (injected in the paw), according to its dilution: (**a**) 10^0^ dilution, (**b**) 10^−1^ dilution, (**c**) 10^−2^ dilution, (**d**) 10^−3^ dilution, and (**e**) 10^−4^ dilution.

The characterization of stages of the infection over time is depicted in Figure 4, after an intradermal injection of the virus, showing a successful model for the disease.

### 3.4. Effects of Bufotenine on Rabies Animal Model

To verify the effects of bufotenine in the animal model of rabies that was previously established (Figure 4), both serum and cells from CNS were evaluated by quantifying the virus titration, as well as through the quantification of anti or pro-inflammatory cytokines and a histological analysis of the brain, as outlined below.

#### 3.4.1. Evaluation of Animal Serum

##### Virus Titration

**Figure 5:**
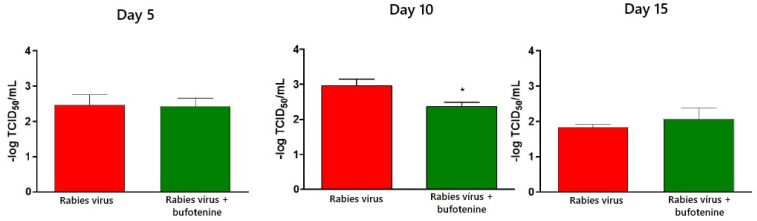
Virus titers on animal serum based on days after inoculation and comparison of inoculation of rabies virus and treatment with bufotenine 5, 10, and 15 days after inoculation. * *p* < 0.01.

Virus titration in mice serum, determined after neutralizing antibody titration using the rapid fluorescent focus inhibition test (RFFIT), was reduced with bufotenine administration after 10 days post-inoculation compared to mice serum without bufotenine (Figure 5).

##### Cytokine Quantification

**Figure 6:**
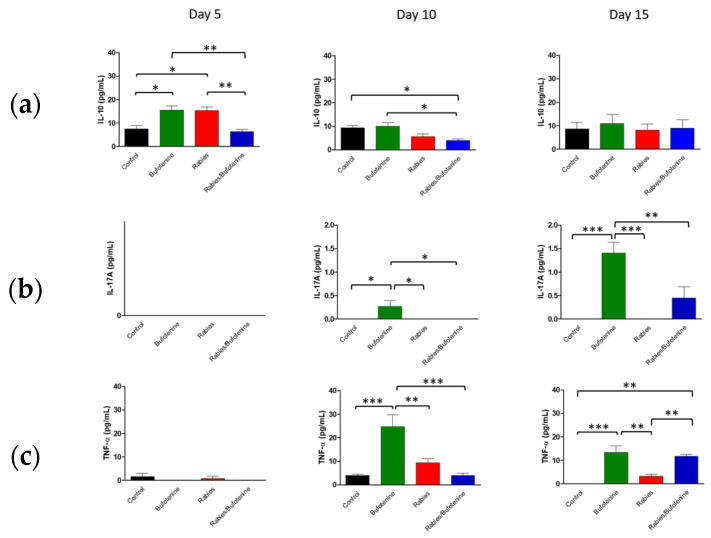

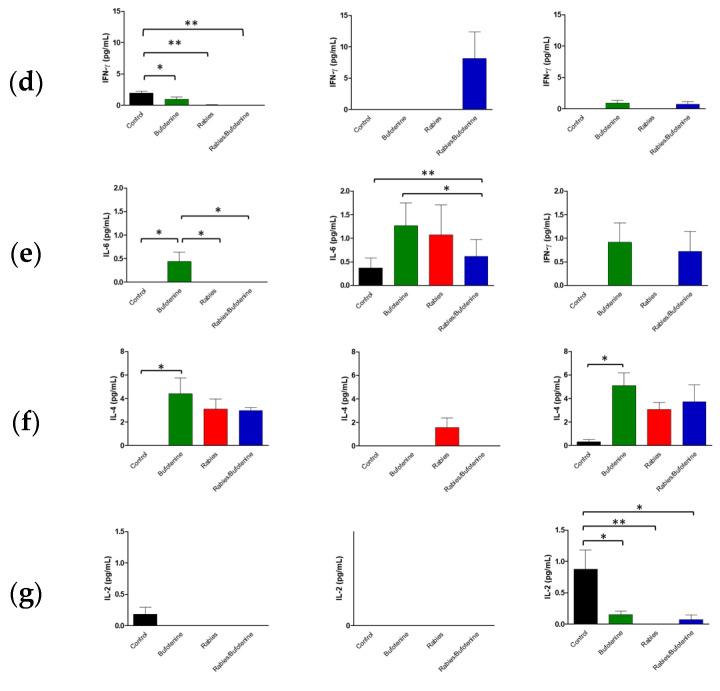
Cytokine quantification in mice serum after rabies virus or rabies virus and treatment with bufotenine. (**a**) IL-10. (**b**) IL-17A. (**c**) TNF-α. (**d**) IFN-γ. (**e**) IL-6. (**f**) IL-4. (**g**) IL-2. * (*p* ≤ 0.05), ** (*p* ≤ 0.01), and *** (*p* ≤ 0.001).

Several cytokines were quantified, as shown in Figure 6. IL-10 was reduced with the treatment with bufotenine after 5 days and maintained 10 and 15 days post-inoculation compared to the rabies group (Figure 6a).

IL-17A was absent in the rabies group and increased 15 days after bufotenine treatment (Figure 6b). Another pro-inflammatory cytokine, TNF-α, decreased 10 days post-inoculation and bufotenine compared to rabies group, but it increased after 15 days (Figure 6c). A peak of IFN-γ was observed in the bufotenine-treated group at day 10 but was reduced after that (Figure 6d). IL-6 has no statistical significance between groups (Figure 6e). IL-4, on the other hand, was reduced after 10 days with bufotenine, while in other days, it remained the same as in the rabies group (Figure 6f). IL-2 has a little increase in day 15 with bufotenine (Figure 6g) without statistical significance.

#### 3.4.2. Evaluation of Animal CNS

##### Virus Titration

**Figure 7:**
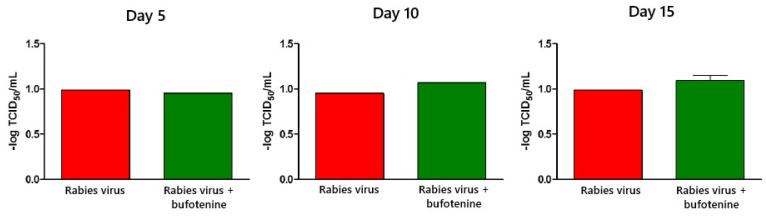
Virus titer on animal CNS according to days after inoculation and comparison of inoculation of rabies virus and inoculation of rabies virus plus treatment with bufotenine 5, 10, and 15 days after inoculation.

Virus titration was also determined in cells (after neutralizing antibody titration) from CNS using the RFFIT. The virus load was not altered with bufotenine treatment and remained the same overtime in both groups (Figure 7). On the other hand, the virus load in the spinal cord was negative in all samples.

##### Cytokine Quantification

When CNS was analyzed, the cytokine quantification pattern was slightly different than the serum.

**Figure 8:**
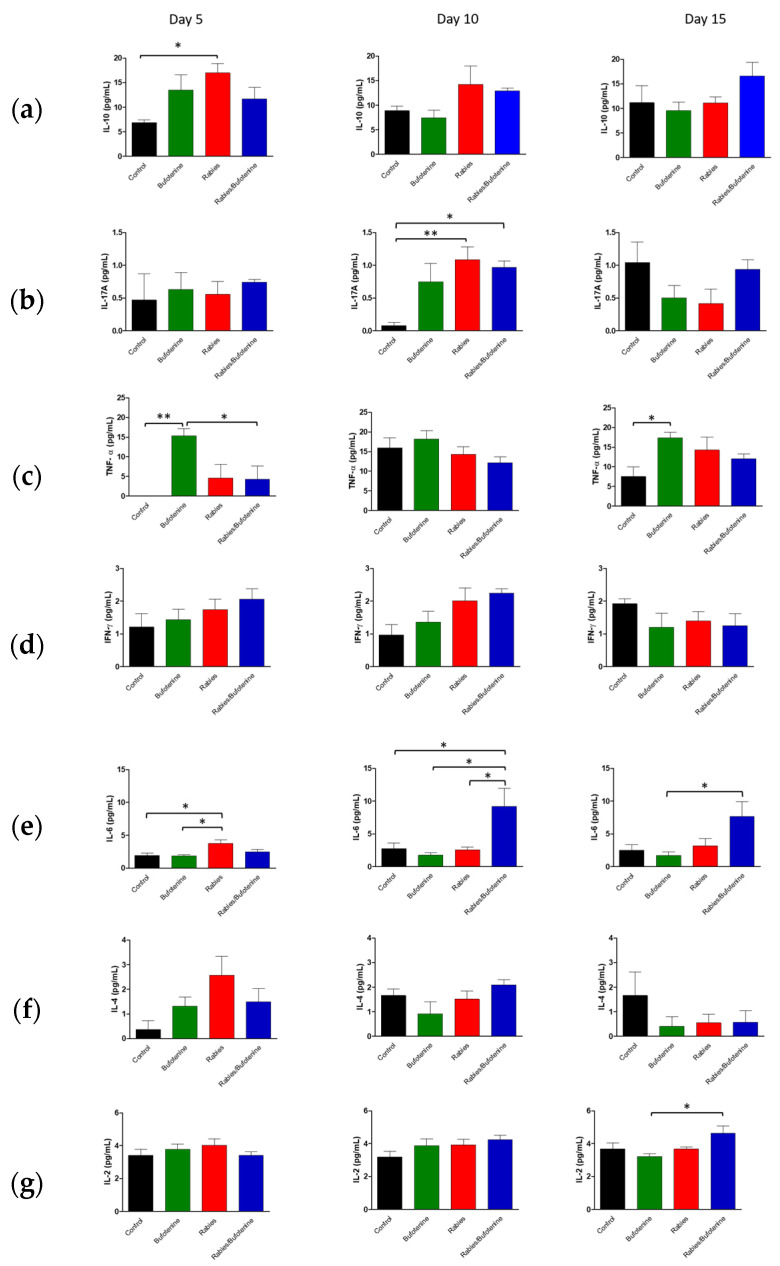
Cytokine quantification in mice CNS after rabies virus or rabies virus plus treatment with bufotenine. (**a**) IL-10. (**b**) IL-17A. (**c**) TNF-α. (**d**) IFN-γ. (**e**) IL-6. (**f**) IL-4. (**g**) IL-2. * (*p* ≤ 0.05), ** (*p* ≤ 0.01).

The IL-10 pattern was similar to the serum (Figure 8a), as well as TNF-α (Figure 8c). On the other hand, IL-17-A was only increased after 15 min in the bufotenine group (Figure 8b).

IFN-γ and IL-2 had no alteration in time and between groups (Figure 8d,g). IL-6 was increased in the bufotenine group in comparison to the only-rabies-injected CNS (Figure 8e). IL-4 levels were reduced over time, mainly in the rabies group, but on day 15, it was similar between groups (Figure 8f).

#### 3.4.3. Histology

**Figure 9:**
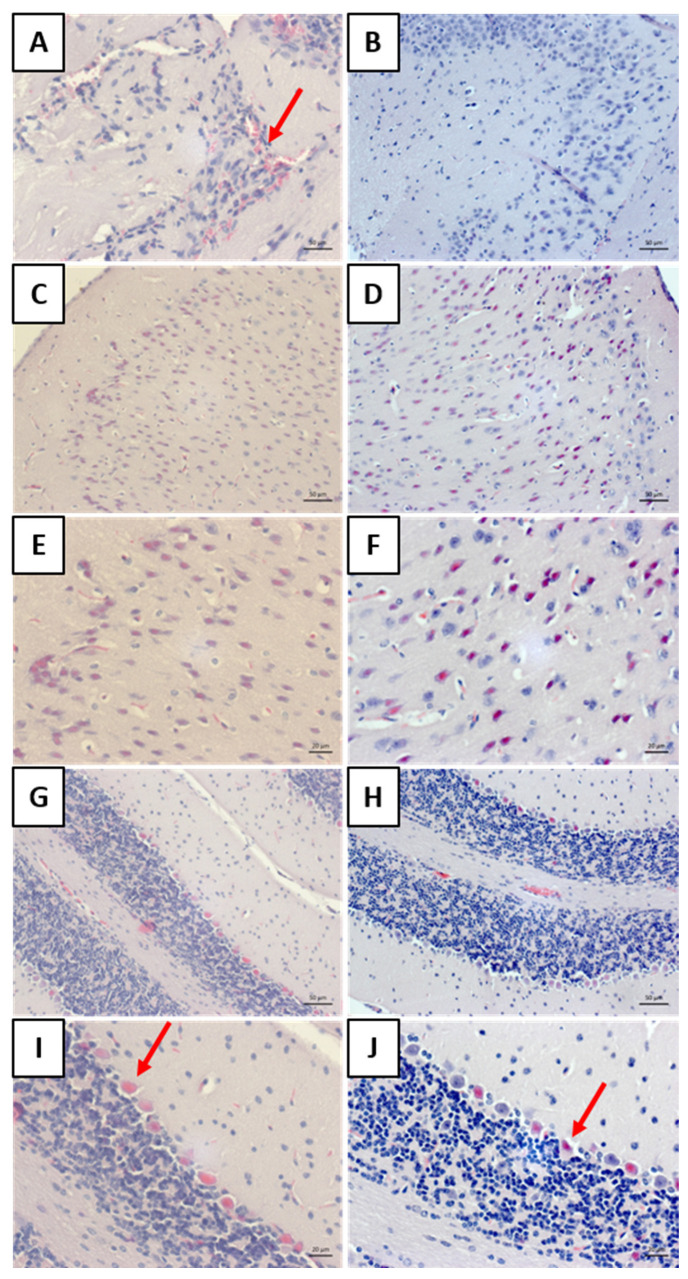
The histology of the CNS stained with hematoxylin and eosin (HE). (**A**) The meningeal region of the rabies group and (**B**) the rabies group treated with bufotenine. The red arrow indicates the region with inflammatory infiltrate. (**C**,**E**) The cortical region of the rabies group. (**D**,**F**) The cortical region of the rabies group treated with bufotenine. (**G**,**I**) The cerebellum of the rabies group. (**H**,**J**) The rabies group treated with bufotenine. The red arrow indicates the presence of anucleate eosinophilic Purkinje cells.

Hematoxylin and eosin (H&E)-stained histological sections revealed the presence of a perivascular mononuclear inflammatory infiltrate in the meninges of the rabies group (Figure 9A), which was not observed in the bufotenine-treated animals (Figure 9B). In the cortex, eosinophilic neurons, suggestive of cellular degeneration, were observed in both groups (Figure 9C,E—rabies group; Figure 9D,F—bufotenine-treated group). In the cerebellum, anucleated and eosinophilic Purkinje cells were identified, also suggesting cellular degeneration. The bufotenine-treated group appeared to have a lower number of eosinophilic cells, although further analyses are required to confirm this finding (Figure 9G,I—rabies group; Figure 9H,J—bufotenine-treated group).

**Figure 10:**
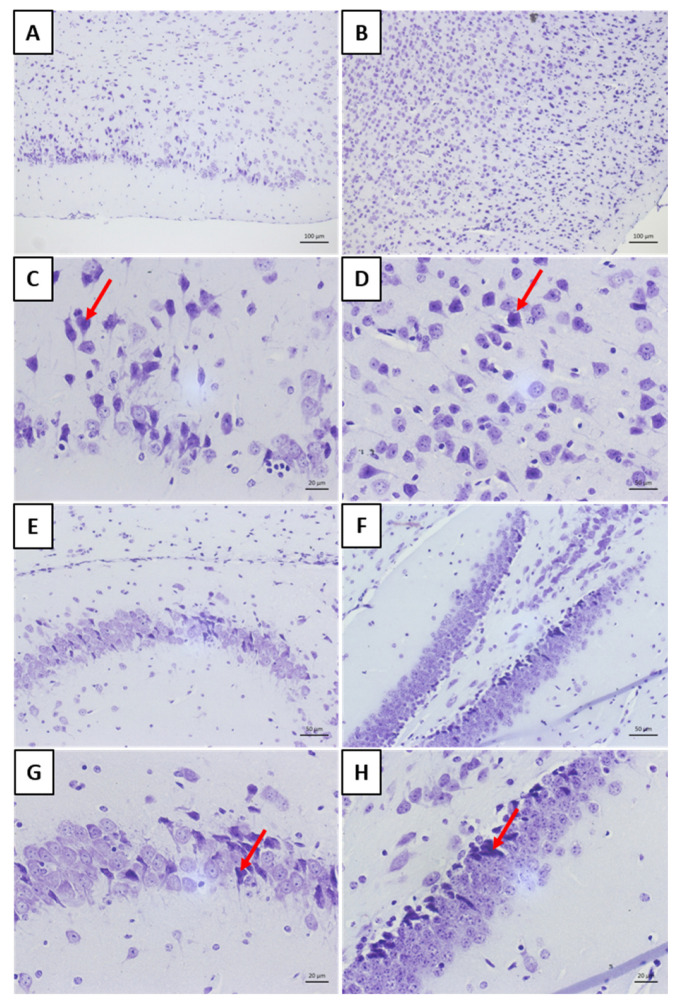
The histology of the CNS stained with cresyl violet. (**A**,**C**) The cortical region of the rabies group. (**B**,**D**) The cortical region of the rabies group treated with bufotenine. (**E**,**G**) The hippocampal region of the rabies group. (**F**,**H**) The hippocampal region of the rabies group treated with bufotenine. The red arrows indicate degenerated neurons.

Cresyl violet staining enabled a more detailed evaluation of neuronal degeneration in the cortical and hippocampal regions (Figure 10). When compared to the bufotenine-treated group, animals with rabies showed a higher number of degenerated neurons (Figure 10A,E,G—rabies group; Figure 10B,D,F,H—bufotenine-treated group). Furthermore, a quantitative analysis revealed a marked reduction in the total number of neurons in the cerebral cortices of rabies-infected animals compared to the bufotenine-treated group (Figure 10A—rabies group; Figure 10B—bufotenine-treated group).

## 4. Discussion

Bufotenine is an alkaloid found in *Anadenanthera colubrina* seed and *Rhinella* species, such as *Rhinella schneideri*. Our group has been studying this molecule for years.

In an in vitro model, we demonstrated that alkaloid inhibited the penetration of rabies virus in mammalian cells in dose- and time-dependent manners [9]. In an in vivo model, with intracerebrally virus-infected mice, bufotenine increased the survival rate from 15 to 40% [11]. It is important to mention that this mouse model used the virus injected directly into the CNS, and now, we present another animal model, administrating the virus in the paw, simulating the course of the disease.

IP1972/16 is a strain compatible with domestic dogs, employed in this study, which matches the characteristics of a wild-type sample, as the proposal is to simulate the accident.

The establishment of street virus strains for conducting the aforementioned in vivo experiments is based on considerations elucidated by Appolinario and Jackson in 2015 [17], which clarified the procedures to be followed when aiming to evaluate the potential of a specific antiviral agent for rabies. Initially, tests must be applied to neuronal cell cultures, and if antiviral activity is confirmed in this context, the experiments should proceed to an in vivo model using fixed and street viruses. The term “street virus” refers to viral isolates from animals that were naturally infected and may present prolonged incubation periods (ranging from 14 days to over 1 year) and variable clinical manifestations [18].

It is known that all mammals are susceptible to rabies; however, certain hosts are considered natural reservoirs as they maintain the virus in circulation within a population. Research conducted in the field of molecular biology has allowed for the identification of genetic differences in rabies virus strains inherent to reservoir species, differentiating them into specific genetic lineages. This, in turn, benefits epidemiological studies on rabies by providing a more grounded analysis that links the disease to the genetic lineage involved [19,20].

It is worth noting that different street genetic lineages also exhibit differences among themselves. A study conducted by Katz et al. (2016) [21] showed that isolates from the hematophagous bat *Desmodus rotundus* had longer incubation times, clinical phases, and replication rates compared to isolates from domestic dogs and tufted capuchin monkeys. Thus, the different tested genetic lineages in rabies studies hold significant importance for comparative analysis.

The titration technique in mice is necessary when the goal is to achieve an expected mortality percentage for conducting a specific assay according to the virus titer. The titration of the wild sample IP1972/16, performed in mice via a plantar pad, enabled the determination of the lethal dose to kill 50% of the population. Based on this result, it was possible to define the number of lethal doses required for the experiment that simulated the progression of a natural rabies virus infection and the administration of bufotenine.

The 50-day observation period for animals used in virus titration is justified by the inoculation site of the sample via plantar pad, which requires a longer time for the virus to travel to the CNS of the animals and cause the disease. The Mouse Inoculation Test (MIT) for rabies virus diagnosis [15]. follows a standard protocol with an observation period of up to 30 days. However, in this case, inoculation is intracerebral, which excludes the virus’s transit time in a natural infection [22,23]. The presentation of data up to the 36th observation day is justified by the lack of changes up to the 50th day of observation during the conclusion phase of this study.

Thus, we could see a faster establishment of infection in groups with dilutions of 10^0^ and 10^−1^, corresponding to the most concentrated virus dilutions. A reduction in the number of animal deaths was observed in the subsequent dilutions. One animal in dilution 10^0^ and dilution 10^−1^ remained alive until the 36th observation day, while all others in these groups were euthanized between days 15 and 18. In this case, factors such as animal immunity, the amount of sample inoculated, and the incubation/observation time should be considered. According to Acha and Szyfres [2], the rabies virus incubation period in humans can vary between 2 and 8 weeks, though in some cases, it can range from 10 days to 8 months and, rarely, even years. Such variations relate to the amount of viruses inoculated during a bite or scratch, the location of the event, the viral load, and the virus strain [21]. The clinical signs of rabies observed in the mice were consistent with a study conducted by Gamon in 2015 [24], which inoculated street samples in isogenic mice through the same route (plantar pad).

No unexpected deaths occurred during the experiment with bufotenine. The experimental design considered using a viral dose that would allow all scheduled euthanasia procedures (on days 5, 10, and 15) to be performed without animal loss, aiming to obtain responses related to the mechanism of action of bufotenine. To this end, the administration of 10 LD_50_ was established, which allowed for the euthanasia of all animals on predetermined days, enabling the collection of the CNS, whole blood, and bone marrow.

The subcutaneous route of administration was selected based on evidence in the literature indicating its association with a lower risk of systemic side effects and a slower absorption rate. This route reduces the need for multiple daily administrations and consequently minimizes stress in organisms already compromised by disease. Moreover, a previous study conducted by our group employed this same route to administer 0.63 mg of bufotenine in 250 µL of NaCl/animal/day in mice intracerebrally inoculated with the rabies virus—a route known to accelerate disease progression. Despite the severity of this model, bufotenine treatment was associated with an increase in survival rate from 15% to 40%.

This study represents the first experimental use of a bufotenine-based solution under conditions simulating accidental infection via the plantar pad. For consistency and comparability, the dosage and route of administration previously established by [11] were employed.

Virus titration was performed using the rapid fluorescent focus inhibition test on animal serum and CVS. In serum, samples showed a statistical difference between the rabies group and the bufotenine group on day 10, with the viral titer being lower in the bufotenine-treated group, suggesting the antiviral action of the alkaloid, and a stronger antibody response compared to the rabies-only group, considering that in the rabies group, a drop in the viral titer occurred between days 10 and 15, characteristic of antibody production on day 15. This drop was not observed in the bufotenine-treated group, suggesting that antibody production becomes more stable starting from as early as day 10. Specific experiments to quantify antibodies will be further performed to confirm this hypothesis.

Histology allowed for the observation of mononuclear cell inflammatory infiltrates around the blood vessels in the meninges of the “rabies” group. This result aligns with other findings on rabies [25,26]. The analysis of the “rabies/bufotenine” group, however, did not demonstrate the presence of this infiltrate. Nevertheless, cell degeneration was observed in both groups, although the “rabies/bufotenine” group seemed to have fewer eosinophilic cells. These results emphasize the action of bufotenine in controlling infection through reducing inflammation by the action of virus infection and activating the immunological system.

The cytokine profiles observed in this study suggest that rabies infection triggers a complex immune response, characterized by early pro-inflammatory activity followed by immunomodulation.

An analysis of central nervous system (CNS) suspensions from isogenic mice revealed a progressive increase in IL-10 levels in the rabies + bufotenine group, contrasting with a gradual decline in the rabies-only (positive control) group. In serum, IL-10 peaked earlier (day 5) in the rabies control group, while the rabies/bufotenine group showed the highest levels at day 15. IL-10 is an immunoregulatory cytokine that suppresses pro-inflammatory cytokine production by activated lymphocytes, shifts immune responses from Th1 to Th2, and enhances B-cell activation [27].

Bufotenine, a serotonin derivative, shares structural similarities with serotonin, which has been shown to mitigate systemic inflammation [28] Given the link between molecular structure and function, bufotenine may exert analogous anti-inflammatory effects, which could potentially explain the elevated IL-10 levels observed.

IL-17A was undetectable in the rabies control serum but appeared on day 15 in the rabies/bufotenine group. In the CNS, IL-17 levels remained more stable in the bufotenine-treated group compared to the rabies-only group. IL-17 is a pro-inflammatory cytokine implicated in autoimmune and infectious diseases and may disrupt blood–brain barrier integrity by downregulating tight junction proteins [29].

TNF was the most highly expressed cytokine in the CNS, with comparable levels between groups. However, in serum, TNF decreased by day 15 in the rabies control group but increased in the rabies/bufotenine group. TNF promotes the production of chemokines, thereby enhancing leukocyte recruitment to inflamed tissues.

IFN-γ expression was similarly reduced in the CNS across all groups, accompanied with low serum levels. IFN-γ is critical for antiviral immunity as it inhibits viral replication and activates macrophages [30]). The slightly better serum response in the rabies/bufotenine group suggests that bufotenine may modulate this pathway.

IL-6 was more prominent in the CNS of the rabies/bufotenine group, while serum expression was detected only in this group on day 15. IL-6 has dual roles, promoting both neutrophil and T-cell activation while also contributing to tissue repair.

IL-4 levels were significant in the CNSs of both rabies-infected groups but higher in the bufotenine-only serum group. This anti-inflammatory cytokine, produced by Th2 cells and granulocytes, modulates lymphocyte activity.

IL-2 was uniformly low in the CNS and undetectable in the rabies control serum but present in the bufotenine-treated group. Its short half-life (<10 min) limits detection during acute injury, though it plays a key role in initiating antigen-specific responses.

## 5. Conclusions

The IP1972/16 virus sample was able to cause rabies in an inoculated mice paw, presenting characteristic symptoms of the disease and proving to be a good model for accidental infection (via the intramuscular route). Moreover, bufotenine appears to mediate, directly or indirectly, the cytokine response in the CNS and serum, in addition to reducing neuronal degeneration caused by rabies at a more advanced stage of the disease.

This study provides compelling evidence that bufotenine, a naturally occurring alkaloid with structural similarities to serotonin, exhibits both antiviral and immunomodulatory effects in experimental rabies infection. Using a street rabies virus strain (IP1972/16) administered via the plantar pad—a model that closely mimics natural infection—we demonstrated that bufotenine treatment reduced viral titers in serum by day 10, suggesting an early antiviral effect. Furthermore, a histopathological analysis revealed that bufotenine attenuated neuroinflammation, as evidenced by the absence of mononuclear infiltrates in the meninges and reduced neuronal degeneration compared to the rabies-only group. Cytokine profiling revealed a distinct immunomodulatory role of bufotenine, characterized by sustained IL-10 elevation, delayed IL-17A and TNF responses, and a more stable IFN-γ profile. These findings suggest that bufotenine may help regulate the immune response, preventing excessive inflammation while maintaining antiviral defenses. The structural resemblance between bufotenine and serotonin further supports its potential as a neuroimmunomodulator, influencing both peripheral and CNS immune dynamics during rabies infection. Despite these promising results, the persistent suppression of IFN-γ underscores the challenges posed by rabies virus immune evasion. Future studies should explore whether bufotenine can enhance survival in conjunction with other antiviral strategies and investigate its impact on antibody production and blood–brain barrier integrity. This work highlights the therapeutic potential of bufotenine in neurotropic viral infections and reinforces the importance of conducting further research into its mechanisms of action.

## Figures and Tables

**Table 1:** Summary of Experimental Groups, Inoculum, and Daily Treatment.

Groups	Inoculum/Route of Administration	
	Accident Simulation (Day 0)	Treatment (Daily)
Saline control	30 µL diluent/plantar pad	250 µL NaCl/subcutaneous
Bufotenine control	30 µL diluent/plantar pad	250 µL NaCl + 0.63 mg bufotenine/subcutaneous
Rabies control	30 µL virus IP1972/16/plantar pad	250 µL NaCl/subcutaneous
Rabies/Bufotenine	30 µL virus IP1972/16/plantar pad	250 µL NaCl + 0.63 mg bufotenine/subcutaneous

## Data Availability

The original contributions presented in this study are included in the article. Further inquiries can be directed to the corresponding author.
